# Looking Back

**Published:** 1904-12-31

**Authors:** 


// The Hospital.

A Journal of

The Medical Sciences and Hospital Administration.

Vol. XXXVII.—No. 953. DECEMBER 31, 1904.

### Looking Back.

The consciousness of doing anything, however simple and familiar, for the last time, can never be wholly without effect even upon the dullest of human imaginations; and, when the action is the writing of a date, it almost compels some retrospect of the period which that date has covered. The figures 1904 will disappear from the front page of our next issue, and the consideration naturally turns our thoughts to the events which the passing year has witnessed. In what respects, and in what degree, does the infancy of the new century fulfil the promise of the old?

The title of this journal naturally directs our thoughts to the influence of the year upon the great and beneficent institutions from which that title is derived; and it would be impossible to deny that definite progress has been made in the direction of rendering their working more economical, and of bringing them into closer touch with the populations by which their immediate services are chiefly required. The operations of the King's Fund, and of the older funds which those operations have stimulated to increased activity, have powerfully tended to promote the utilisation, by every hospital, of the experience of its friendly rivals; and the light recently thrown upon leakages, some of which at least may be avoidable, will inevitably bring about improvements in all. The recent suggestion that the hospitals should combine to establish a common system of buying, and thus to obtain the full advantages of the cheapest markets for their daily wants, is one which appears not to be destitute of promise, and would be calculated, if guided by sufficient commercial knowledge and experience, to bring about considerable diminutions of expenditure in matters of the first necessity. The tendency of the managers of the Fund to exert pressure for bringing about the amalgamation of separate hospitals is one which will receive the approval of all who have at heart the true interests of the London hospitals and of the sick poor; and the realisation of the views expressed in the annual report of the King's Fund as to the further amalgamation of certain institutions is likely to result in a considerable saving of money, for one of the disadvantages of the present profusion of special hospitals is the inevitably increased expenditure. Another of the serious evils which the injudicious multiplication of small or special hospitals involves is its tendency to withdraw from the medical schools the materials essential to teaching. There can be little question that such branches of surgery as ophthalmology and otology suffer in this way, and that the profession generally would be better instructed with regard to them if the diversion of patients to special hospitals could be restrained.

Another highly important question which has come into fresh prominence during the year, although it is of very old standing, is that of the relation of hospitals to the very considerable number of general practitioners who reside in districts containing large industrial populations. Medical men so circumstanced are accustomed, as a rule, to adjust their fees and their methods of receiving them to the financial position of their neighbours, and are well aware that many persons who wear soiled clothing and display unwashed countenances, are perfectly well able to pay the moderate charges for medical attendance which are customarily made to them. They are equally well aware that a large proportion of such people habitually resort to the nearest hospital, and obtain advice and medicines either gratuitously in that sense of the word which means "granted without claim or merit," or at the expense of some small payment, which is described as "nominal" by the hospital authorities, but which is regarded by those who pay it as a full return for the benefits which they receive, and also as an evidence that the neighbouring "doctors" habitually overcharge them. To whatever extent these conditions exist, they constitute an evil which tends to grow and increase. The medical men of the locality lose income, the patients lose manliness and self-respect, the money of subscribers is misapplied, the really poor are excluded, and the only persons deriving benefit are the brewers and publicans of the locality. The question how to guard against abuses of this description is one in which the supporters of hospitals and the members of the medical profession should be equally interested, and should be prepared to meet each other half-way; but, unfortunately, this harmony has not always prevailed. The East London Medical Society has recently approached the authorities of the London Hospital upon the question, and has done so, we think, in a very temperate and judicious manner; but it can hardly be maintained that their representations have been treated with the respect which they deserve. The hospital authorities do not appear to have fully realised that they were asked to co-operate in the correction of an abuse mainly of their own creation, and to some extent inseparable from the work which they were carrying on, and they have displayed an inclination either to resent the appeal of the society, or, at all events, to show that it was unnecessary or unfounded. In such an endeavour they are not likely to be successful; and it would be more judicious to unite with the applicants in an endeavour to ascertain the whole truth of the matter, and to apply such remedies as the facts, when fully disclosed, might be found to require. In the meanwhile the business of investigation and of protest is too large a one to be undertaken unaided by a local medical society, even although it numbers among its members the practitioners who are chiefly aggrieved.

